# The Relationship of Fat Distribution and Insulin Resistance with Lumbar Spine Bone Mass in Women

**DOI:** 10.1371/journal.pone.0129764

**Published:** 2015-06-11

**Authors:** Francisco J. A. de Paula, Iana M. de Araújo, Adriana L. Carvalho, Jorge Elias, Carlos E. G. Salmon, Marcello H. Nogueira-Barbosa

**Affiliations:** 1 Department of Internal Medicine, Ribeirao Preto Medical School, USP, Ribeirão Preto, Brazil; 2 Department of Physics, Faculty of Philosophy, Sciences and Arts of Ribeirao Preto, USP, Ribeirão Preto, Brazil; Virgen Macarena University Hospital, School of Medicine, University of Seville, SPAIN

## Abstract

Bone marrow harbors a significant amount of body adipose tissue (BMAT). While BMAT might be a source of energy for bone modeling and remodeling, its increment can also represent impairment of osteoblast differentiation. The relationship between BMAT, bone mass and insulin sensitivity is only partially understood and seems to depend on the circumstances. The present study was designed to assess the association of BMAT with bone mineral density in the lumbar spine as well as with visceral adipose tissue, intrahepatic lipids, HOMA-IR, and serum levels of insulin and glucose. This cross-sectional clinical investigation included 31 non-diabetic women, but 11 had a pre-diabetes status. Dual X-ray energy absorptiometry was used to measure bone mineral density and magnetic resonance imaging was used to assess fat deposition in BMAT, visceral adipose tissue and liver. Our results suggest that in non-diabetic, there is an inverse relationship between bone mineral density in lumbar spine and BMAT and a trend persists after adjustment for weight, age, BMI and height. While there is a positive association between visceral adipose tissue and intrahepatic lipids with serum insulin levels, there is no association between BMAT and serum levels of insulin. Conversely, a positive relationship was observed between BMAT and serum glucose levels, whereas this association was not observed with other fat deposits. These relationships did not apply after adjustment for body weight, BMI, height and age. The present study shows that in a group of predominantly non-obese women the association between insulin resistance and BMAT is not an early event, as occurs with visceral adipose tissue and intrahepatic lipids. On the other hand, BMAT has a negative relationship with bone mineral density. Taken together, the results support the view that bone has a complex and non-linear relationship with energy metabolism.

## Introduction

Only recently has the mutual influence between bone and peripheral insulin-sensitive tissues (muscle and adipose) started to receive proper attention[1]. Initial studies recognized that both lean and fat body mass have a positive relationships with bone mass, suggesting that not only muscle but also adipose tissue potentially have beneficial effects on the skeleton[2]. This perception has been reconsidered with the acquisition of new data showing that obesity does not protect bone from fracture[3]. In parallel, new evidence has emerged revealing a complex network linking bone to energy metabolism [4]. In this new scenario, endocrine molecules originating in adipose tissue (i.e., leptin and adiponectin) as well as in bone (osteocalcin) are active factors able to modulate bone remodeling and intermediary metabolism, respectively[4,5].

The distribution of fat within the body has a great impact on the emergence of metabolic and cardiovascular disorders such as insulin resistance, diabetes mellitus, dyslipidemia and arterial hypertension. Visceral adipose tissue (VAT) is not only less sensitive to insulin, but also has a greater potential to generate insulin resistance than subcutaneous adipose tissue (SAT). The spillover of free fatty acids (FFAs) derived from the increased lipolytic activity in VAT leads to an accumulation of fat in liver and muscle[6]. Ectopic fat in liver [intrahepatic lipids (IHL)] and muscle is directly involved in functional (insulin resistance) and structural abnormalities [nonalcoholic fatty liver disease (NAFLD)[7]. As expected, there is great interest in the study of the effects of adipose tissue distribution on bone mass. In this respect, bone is unique in that it harbors its own type of adipose tissue, referred to as bone marrow adipose tissue (BMAT). Moreover, it was recently observed that BMAT is an important source of adiponectin, the sole molecule able to increase insulin sensitivity[8]. Under these circumstances, further investigation is necessary to clarify several points linking insulin resistance to bone mineral density (BMD) and BMAT.

The increment in BMAT has been observed in several physiological conditions and diseases associated with osteoporosis, including aging, menopause, and glucocorticoid-induced osteoporosis, all having increased insulin resistance in common[9]. On the other hand, BMAT has also been found to be increased in other disorders associated with osteoporosis in which insulin resistance is not present, namely anorexia nervosa[10]. Therefore, while VAT and IHL are closely linked to insulin resistance, the association of insulin resistance with BMAT and BMD still is to be clearly elucidated. Our results supported previous data showing that BMD is negatively correlated with BMAT. However, no association was observed between BMAT and HOMA-IR. Moreover, there was no relationship between BMAT and VAT or IHL

## Subjects and Methods

### Subjects

A prospective cross-sectional study was conducted on 31 non-diabetic women. In addition, none of the volunteers met any of the other exclusion criteria (kidney and liver disease, use of diabetogenic or steatogenic drugs, as well as medication with osteomineral influence). The study was approved by the institutional review board of the University Hospital, Ribeirao Preto Medical School, USP (protocol number 211/2012). All volunteers gave written informed consent.

A blood sample was drawn under standard conditions between 8 and 9 am after an overnight fast. The biochemical measurements (albumin, glucose, calcium, phosphorus, alkaline phosphatase, cholesterol, HDL-cholesterol and triglycerides) were immediately performed using an autoanalyzer (CT 600i, Wiener Lab, Buenos Aires, Argentina). A blood sample for HbA1c measurement was collected into EDTA containing tubes. HbA1c was measured using a fully automated BioRad D-10 HPLC analyzer (Bio-Rad Laboratories Ltd., USA) immediately after collection. The serum samples used for the other parameters (IGF-I, insulin, iPTH, 25-hydroxyvitamin D) were processed and kept frozen at -80°C until assessment. The chemiluminescence method was used to measure IGF and iPTH (Immulite I, Siemens, Los Angeles, CA, USA) and 25-hydroxyvitamin D (25-OHD) (Liaison, Diasorin, Saluggia, Italy). All determinations had intra- and interassay errors below 10%.

### Image evaluation

Bone mineral density in the lumbar spine (L1-L4), femoral neck, total hip, 1/3 radius and total body was determined by dual-energy X-ray absorptiometry (Discovery Wi, QDR series, Hologic, Waltham, MA, USA). The precision error was 1.2%, 2.3%, 2.7%, 1.7% for L1-L4, femoral neck, total hip and 1/3 radius, respectively. BMD values are reported as g/cm^2^ and T-Score. The equipment is calibrated daily with a phantom provided by the manufacturer. The *in vivo* precision error for lumbar spine assessment was 2.0%.

#### Magnetic resonance imaging acquisition and analysis

All volunteers underwent spine and abdominal MRI on a 1.5T system (Philips ACHIEVA, Philips Medical Systems; Best, Netherlands). For lumbar spine spectroscopy acquisition we used a sagittal T2 weighted fast spin echo (FSE) sequence as a reference for the placement of a 1.5 x 1.5 x 1.5 cm^3^ voxel in the center of the third lumbar (L3) vertebral body. The 1H-MRS, proton magnetic resonance spectroscopy was performed by the Point Resolved Spectroscopy (PRESS) technique and the spectroscopy parameters were: echo time (TE) = 40/60/80 ms, repetition time (TR) = 2000 ms, 8 average, without fat suppression, 1 minute duration. The data were processed with LC Model software and the values obtained were used to calculate the water and fat fractions [11].

The abdominal MRI protocol was performed using a phased-array torso coil and the following sequences were obtained: 1) a coronal turbo-spin-echo (TSE) T2-weighted sequence with breath-holding used as a locator; 2) breath-holding axial gradient double-echo T1-weighted sequence, in-phase (echo time = 4.2 ms) and out-of-phase (echo time = 2.1 ms), slice thickness = 6 mm, with acquisitions in the upper abdomen including the liver and in the lower abdomen with center on the umbilical region. Liver steatosis was evaluated objectively by obtaining the fat fraction as described by Fishbein et al.[12]. The pair of in- and out-of- phase images at the corresponding level of the main portal vein was used to measure signal intensity (SI), which was obtained using region of interest (ROI) measurements in four representative segments of the liver including anterior and posterior right lobe and medial and lateral left lobe segments. ROIs were placed in areas devoid of blood vessels, motion artifacts or partial volume effects. The ROI areas were constant for each segment in the intra-individual sequences. Mean SI levels for each ROI were recorded for each liver segment evaluated and an average SI of all segments was then calculated for each image. The hepatic fat fraction was calculated from the average SI data for each image using the formula: fat fraction = (SIn-phase−SIout-of-phase)/2SIin-phase.

The analysis of visceral and subcutaneous fat was performed with the Display software developed by the Brain Imaging Center of the Montreal Neurological Institute (http://www.bic.mni.mcgill.ca/software/Display/Display.html) using semiautomatic segmentation at the level of the umbilicus. All areas of both compartments were recorded in mm^2^ and total abdominal fat was recorded as the sum of visceral and subcutaneous fat.

### Statistical analysis

Linear (and nonlinear) regression models in a simple and multiple approaches were performed. In the multiple models, age, weight, height, BMI and HOMAIR were considered as covariates. Specifically for the associations between VAT and BMAT, IHL and HOMA-IR we introduced quadratic parameters in the models, resulting in a non-linear relationship between the variables. All analyses were carried out using SAS 9.3 software (SAS Institute Inc., SAS/STAT User’s Guide, Version 9.3, Cary, NC: SAS Institute Inc., 2010). The level of significance was set at 0.05.

## Results

Table 1 shows the clinical characteristics of the subjects. The study included 31 women. Their age ranged from 21 to 68 years (median = 54 years), weight from 42.8 to 97.8 Kg (median = 65.7 Kg), height from 146 to 180 cm (median = 1.62 m), and BMI from 17.5 to 37.3 Kg/m^2^ (median = 25.0 Kg/m^2^). The rate of individuals of normal weight was 48.4% (n = 15), while 32.2% (n = 10) and 19.4% (n = 6) were classified as overweight and obese according to BMI, respectively. Individual values are showed in supporting information (S1 Table).

**Table 1:** Clinical characteristics of the subjects.

Women (n = 31)	Mean±SD
Age (years)	47.8±14.8
Weight (Kg)	67.3±11.8
Height (m)	1.62±0.08
BMI (Kg/m^2^)	25.5±4.9
Glucose (mg/dL)	88.9±6.5
HbA1c (%)	5.5±0.4
25-OHD (ng/mL)	23.5±9.5
HOMA-IR	2.06±1.17

BMI = body mass index; HbA1c = glycated hemoglobin A1c; 25-OHD = 25-hydroxyvitamin D and HOMA-IR = homeostatic model assessment- insulin resistance.

All subjects had glucose and HbA1c values below the cutoff for the diagnosis of diabetes mellitus. However, 1 individual exhibited serum glucose levels of 105 mg/dl. In addition to this volunteer, 10 other subjects showed HbA1c values ranging from 5.7 to 6.5%, representing a pre-diabetes condition. HOMA-IR index ranged from 0.79 to 5.59, with a mean level of 2.06±1.17. The mean serum level of 25-OHD was 23.5±9.5 ng/ml. Fifty-two percent of the subjects showed serum 25-OHD levels above 20 ng/ml, normal according to the Institute of Medicine. All individuals exhibited serum creatinine levels in the normal range.

**Fig 1:**
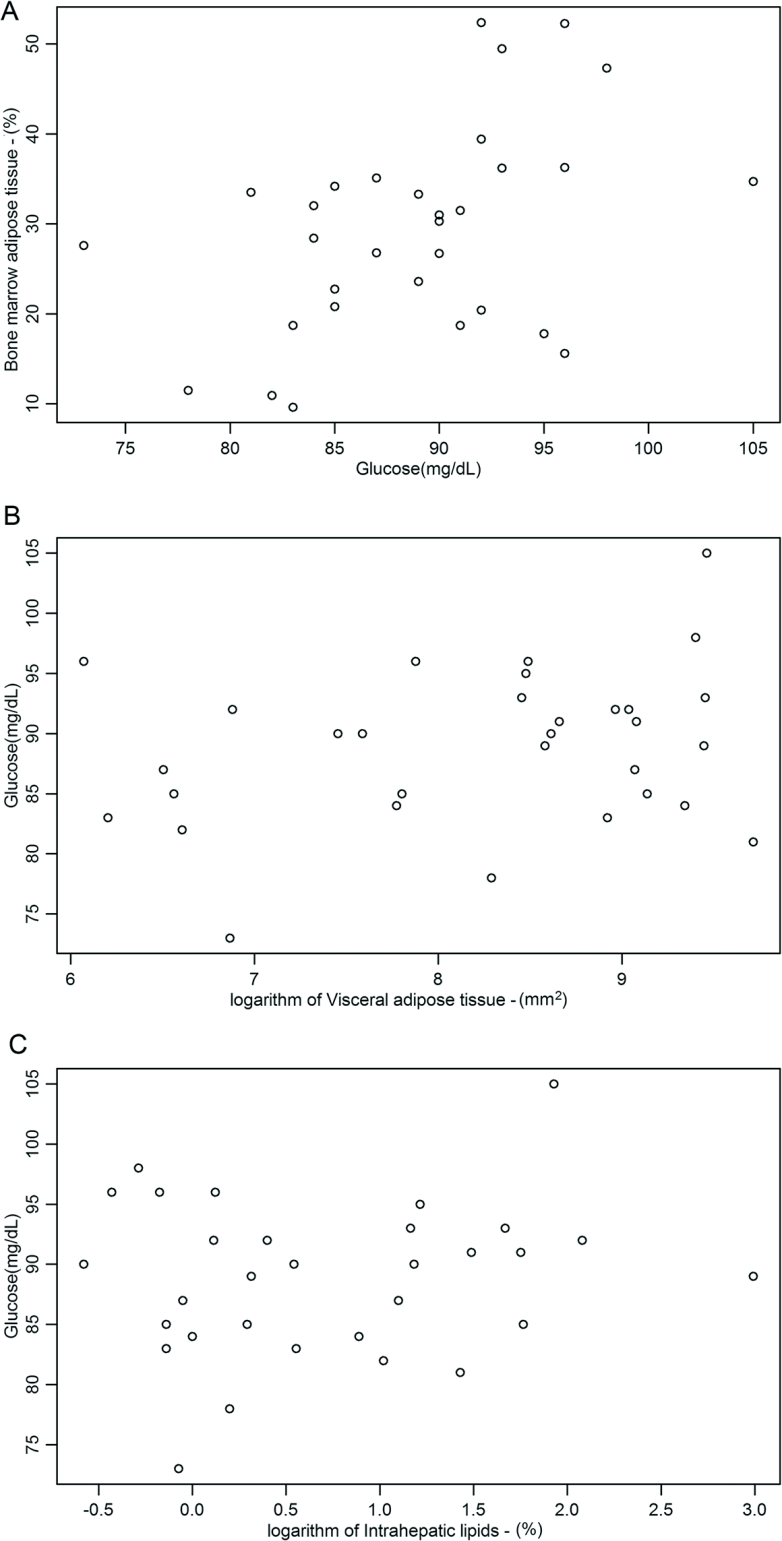
Association between bone marrow adipose tissue (BMAT) (A), visceral adipose tissue (VAT) (B) and intrahepatic lipids (IHL) (C) with serum levels of glucose.

The mean values of BMAT, VAT and IHL for the group as a whole were 29.3 ± 11.5%; 5803 ± 4574 mm^2^, IHL = 3.12 ± 3.71%, respectively. There was no association between BMAT and weight. Also, no association was observed between BMAT and HOMA-IR (*p* = 0.97; r^2^ = 0.0001) or serum levels of insulin (*p* = 0.90; r^2^ = 0.01) (Table 2). However, BMAT was associated with serum glucose (Fig 1A) levels (*p* = 0.01; r^2^ = 0.19) of and HbA1c values (*p* = 0.02; r^2^ = 0.17). These relationships did not apply after adjustment for age, body weight, BMI and height (Table 2). There was no relationship between VAT and serum levels of glucose (Fig 1B), as well as between IHL and serum levels of glucose (Fig 1C) and Table 2.

**Table 2:** Linear and non-linear regression model analysis: associations of BMAT, BMD, VAT and IHL with metabolic parameters.

	Model 1					Model 2				
Associations	Estimate	p-value	IC 95%		r^2^	Estimate	p-value	IC 95%		r^2^
BMAT x weight (A)	0.12	0.52	-0.25	0.48	0.01	-0.31	0.07	-0.65	0.03	0.57
BMAT x HOMAIR (B)	0.08	0.97	-3.76	3.92	0.0001	0.26	0.86	-2.76	3.27	0.58
BMAT x Insulin (B)	-0.54	0.90	-8.93	7.85	0.001	1.31	0.68	-5.2	7.81	0.58
BMAT x Glucose (B)	0.78	0.01	0.17	1.38	0.19	0.27	0.27	-0.22	0.77	0.62
BMAT x HbA1c (B)	10.63	0.02	1.60	19.66	0.17	-0.88	0.87	-11.63	9.86	0.60
*BMAT x VAT (C)	37.11	0.21	-22.79	97.00	0.27	-4.57	0.84	-52.68	43.52	0.66
BMAT x IHL (C)	2.21	0.36	-2.68	7.12	0.03	0.36	0.89	-5.34	6.08	0.57
BMD x BMAT (C)	-0.004	0.03	-0.007	-0.0002	0.14	-0.005	0.07	-0.01	0.0003	0.40
BMD x VAT (C)	-0.0003	0.99	-0.04	0.04	0.00001	0.02	0.56	-0.05	0.08	0.30
BMD x IHL (C)	0.003	0.91	-0.04	0.05	0.0005	0.02	0.59	-0.05	0.09	0.30
*HOMAIR x VAT (B)	-4.73	0.13	-10.9	1.43	0.28	-4.05	0.26	-11.32	3.22	0.31
HOMAIR x IHL (B)	0.76	< 0.01	0.21	1.3	0.24	0.55	0.12	-0.16	1.27	0.29
Glucose x VAT (B)	1.53	0.16	-0.62	3.7	0.07	0.22	0.91	-3.54	3.98	0.23
Glucose x IHL (B)	1.1	0.42	-1.67	3.87	0.02	0.54	0.74	-2.83	3.92	0.22
Insulin x VAT (B)	2.13	0.03	0.28	3.98	0.18	2.12	0.19	-1.12	5.37	0.26
Insulin x IHL (B)	3.51	0.01	0.86	6.16	0.22	2.61	0.13	-0.87	6.09	0.28
*IHL x VAT (B)	-2.52	0.24	-6.81	1.78	0.35	-2.66	0.25	-7.35	2.03	0.45

(A) adjustment for age, HOMA-IR and height;

(B) adjustment for age, weight and BMI;

(C) adjustment for age, weight, BMI and HOMA-IR;

*The model includes a quadratic term [log(VAT)]^2^, suggesting a non-linear relationship.

**Fig 2:**
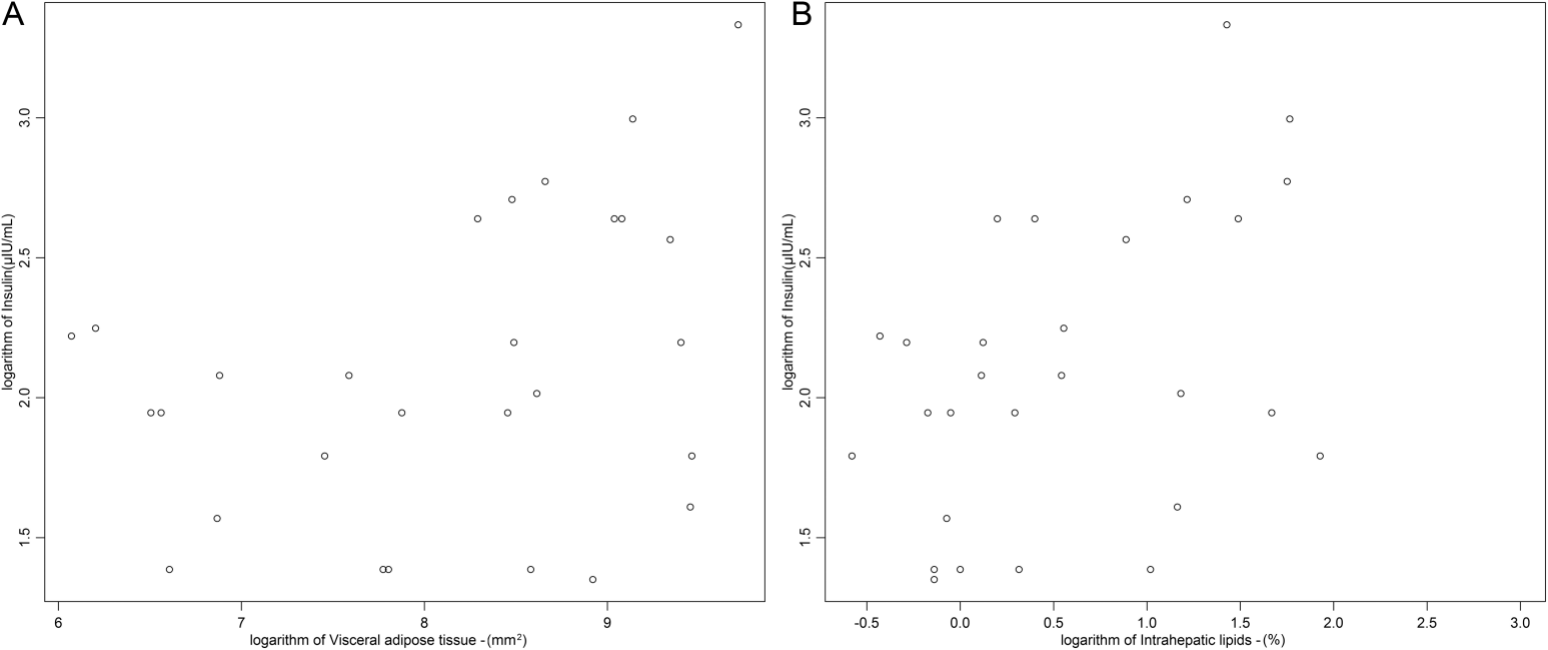
Association between visceral adipose tissue (VAT) (A), intrahepatic lipids (IHL) (B) with serum levels of insulin.

VAT was not associated with IHL (*p* = 0.24; r^2^ = 0.35) (Table 2). VAT (*p* = 0.03, r^2 =^ 0.18) (Fig 2A) and IHL (*p* = 0.01, r^2^ = 0.22) (Fig 2B) had a direct relationship with serum levels of insulin and (Table 2). In addition, IHL showed relationship with HOMA-IR (*p*< 0.01, r^2^ = 0.24), Table 2. These relationships were weakened after adjustment for age, weight, BMI and height, losing statistical significance. No significant correlation was observed between BMAT and VAT (Table 2).

**Fig 3:**
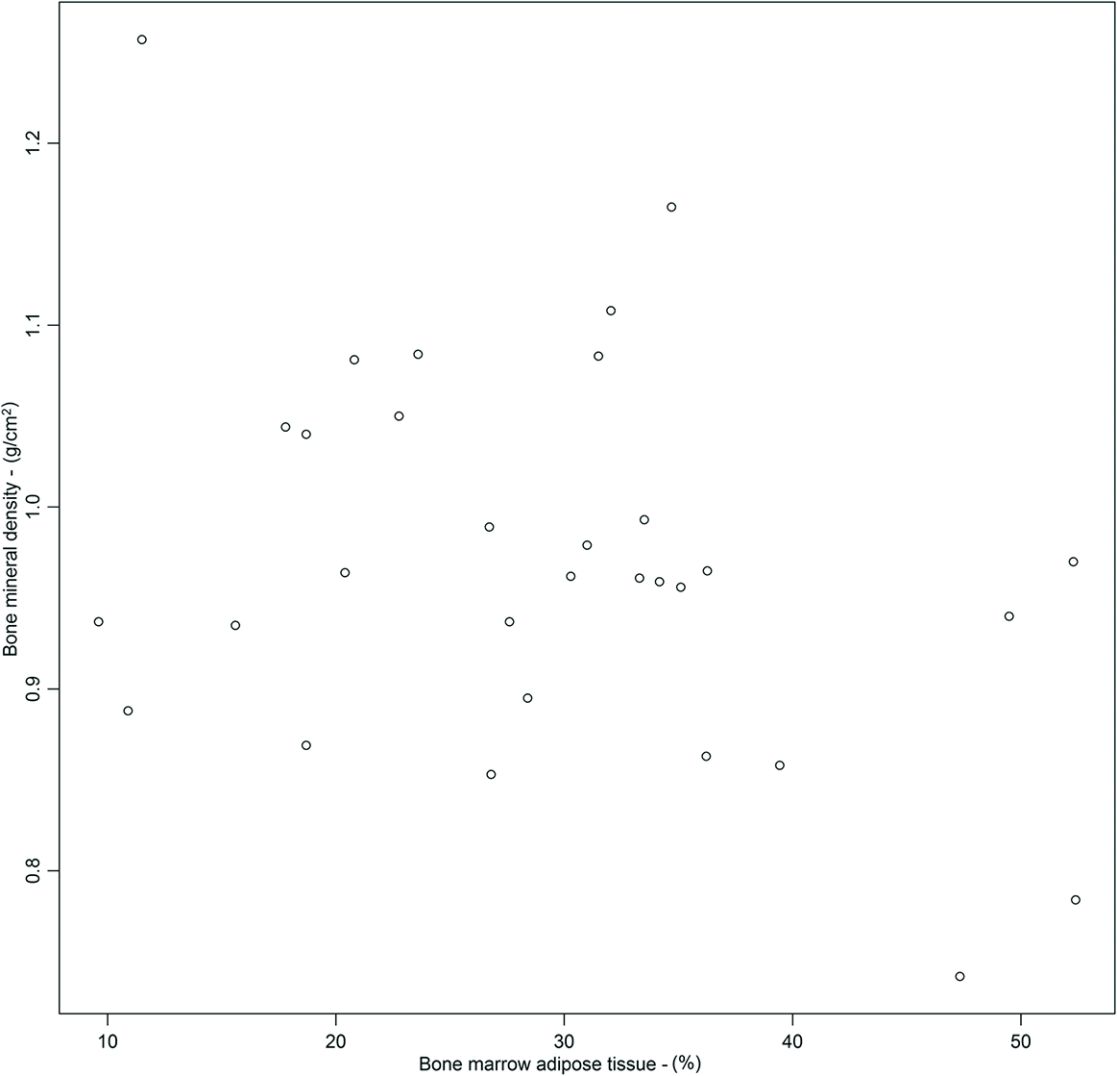
Association between bone mineral density (BMD) with bone marrow adipose tissue (BMAT).

Lumbar spine BMD was negatively associated with BMAT (*p* = 0.03; r^2^ = 0.14) (Fig 3). After adjustment for age, weight, BMI and height the association lost strength (*p* = 0.07, r^2^ = 0.40). On the other hand, there was no relationship between BMD with VAT and IHL (Table 2).

## Discussion

Our data indicated that, unlike the deposit of lipids in VAT and liver, lipid deposits in BMAT are not correlated with insulin resistance in women. In addition, while BMAT has a negative relationship with bone mass, VAT and IHL are not associated with bone mass in adult non-diabetic women.

The detrimental effects of obesity, especially central obesity, on energy metabolism and the cardiovascular system have been well established. Also, it is well known that body weight is an important determinant of BMD, meaning that low body weight is a risk factor for osteoporosis[13]. Unexpectedly, prospective studies performed in the last five years have shown that obese individuals are not protected from fracture[3,14]. Therefore, great attention has been directed at the role of adipose tissue distribution in BDM. In this case, not only VAT, but also BMAT matters. BMAT was previously considered to be merely a filler of bone marrow space as the requirements for hematopoiesis decreases. Indeed, BMAT potentially is a key player in bone homeostasis since marrow adipocytes and osteoblasts have the same mesenchymal stem cells in common. Moreover, BMAT might be a source of both the energy required for bone anabolism and the production of adipokines that induce a negative imbalance in bone remodeling.

In the present study including pre- and postmenopausal, nondiabetic, predominantly nonobese women it was observed that lumbar spine BMD has an inverse relationship with BMAT. These data support previous studies indicating that increased BMAT is a marker of osteopenia in individuals with anorexia nervosa, obesity, and hypercortisolism[15,16,17].

Differently from the present study, Russell et al observed that VAT is a negative predictor of spine BMD[18]. Curiously, Russell et al also found that adiponectin, a molecule that decreases with weight gain, is a negative predictor of BMD, while leptin, an adipose tissue-dependent factor, is a positive predictor of BMD[18]. The results of the present study are not directly comparable to those obtained by Russell et al because the impact of body weight gain on bone mass in adolescent girls seems to be different from its effects in adults. A cohort study performed in the United Kingdom showed that the positive relationship between body weight and bone mass is initially attenuated and subsequently reversed during puberty[19]

The results of the present study highlight the differences in the relationship between bone and adipose tissue in predominantly normal and overweight women in comparison with previous data obtained in obese women[17]. Bredella et al observed an inverse association between VAT and BMD in obese premenopausal women (mean BMI = 36.7±4.2 kg/m^2^). In that study quantitative computed tomography (QCT) was used to assess body composition and lumbar trabecular BMD[17]. In another study by the same group, the associations between ectopic fat and bone marrow fat was investigated in obese men and women (BMI = 33.1 kg/m^2^)[20]. In this condition, the authors found a weak, but significant, positive association of intramyocellular lipids and IHL with BMAT. Although insulin resistance was evaluated in that study, the authors did not mention the association between IR and BMAT. In the present study it was observed for the first time that HOMA-IR values are not positively correlated with BMAT in predominantly normal and overweight women. This suggests that such occurrences are a late manifestation in comparison to the emergence of insulin resistance in VAT and liver. In contraposition, Hanley et al observed in a cross-sectional study of over 7000 individuals from Canada that BMD is increased in type 2 diabetes mellitus. Also, they observed that the association remains even after adjustment for confounding parameters. Hanley et al suggested that their results reflected previous anabolic effects of insulin on bone[21].

Previous studies have shown that hyperglycemia may have detrimental effects on bone. For instance, it was observed that blood glucose levels were associated with bone resorption in type 1 diabetes mellitus [22]. In animal models of type 2 diabetes mellitus (WBN/Kob rats), the appearance of hyperglycemia coincides with the increase of non-enzymatic cross-links (pentosidine) and the reduction of bone strength[23]. In support of these data, there is also evidence of the effects of advanced glycation end products as mechanisms for the emergence of osteoporosis in clinical investigations[24]. In the present study we observed a positive correlation of HbA1c and serum glucose levels with BMAT, suggesting another potential mechanism for bone weakening related to hyperglycemia. However, the association vanishes after taken into account confounders’ factors such as weight and age. These results call attention to the complex network that exists between bone and energy metabolism and to the circumstantial association of mechanisms linking bone fragility to disorders associated with insulin resistance. Four ingredients depict the heterogeneity and conditionality of this association: a) osteoblasts harbor insulin receptors and their deletion leads to osteopenia [25]; b) there are results showing that VAT has a negative relationship with BMD in obese individuals[17]; c) an exogenous activator of PPAR-γ (e.g. rosiglitazone) induces both increases in BMAT and decreases in VAT, but at the same time reduces BMD and enhances insulin sensitivity[26], and d) low body weight, a condition usually associated with higher insulin sensitivity than obesity, is also implicated in impairment of bone mass development and maintenance[27].

The present study has limitations, including the small sample size. In addition, the cross-sectional design prevents the determination of causality between the targeted parameters. On the other hand, the techniques used to estimate lipids at distinct sites were appropriate, representing a fundamental point for the acquisition of original results about the differential relationship between insulin resistance and fat deposition in diverse tissues, especially bone.

In conclusion, the present study shows that lumbar spine BMD in adult non-diabetic women has a specific relationship with fat distribution, insulin resistance and serum glucose levels. While lumbar spine BMD exhibited an inverse relationship with BMAT, it was not associated with VAT and IHL, or with HOMA-IR. Conversely, BMAT showed a positive association with fasting serum glucose levels, which was weakened after adjustment for age, body weight, height and BMI. Taken together, these results highlight that the relationship between bone and energy metabolism is complex and circumstantial, likely depending on age, gender and weight. Genetic heterogeneity may contribute to reporting of wide variation in skeletal and BMAT responsiveness to metabolic and hormonal stimuli. Nevertheless, longitudinal studies in conditions associated with insulin resistance (i.e., obesity and hypercortisolism) as well as with improvement in insulin sensitivity (i.e., exercise and weight loss) certainly will allow the elucidation of important points on the complex network involving bone cells, adipose tissue and insulin resistance.

## Supporting Information

S1 Table (PDF)
