# Supplementary material for: The Relationship of Fat Distribution and Insulin Resistance with Lumbar Spine Bone Mass in Women
Source: PLoS One. 2015 Jun 11;10(6):e0129764. doi: 10.1371/journal.pone.0129764 (PMC4466243; doi:10.1371/journal.pone.0129764)
Supplement: S1 Table — (PDF) [file pone.0129764.s001.pdf]

Table S1- Raw data: clinical characteristics, biochemical assessment, bone mineral density (BMD), bone marrow adipose tissue (BMAT), visceral adipose tissue (VAT), HOMA-IR and intrahepatic lipids (IHL)

	Age	Height	Weight	BMI	Glucose	HbA1c	BMD	BMAT	VAT	Insulin	HOMA-IR	IHL
1	21	1.792	65.7	20.5	85	5.5	1.081	20.8	707	7.0	1.47	1.34
2	22	1.69	58.7	20.6	83	5.5	0.937	9.6	494	9.5	1.94	1.74
3	23	1.565	42.8	17.5	87	4.6	0.853	26.8	668	7.0	1.50	0.95
4	28	1.52	49.4	21.4	82	5.1	0.888	10.9	740	4.0	0.81	2.77
5	28	1.62	63.0	24.0	78	5.3	1.257	11.5	3986	14.0	2.69	1.22
6	29	1.63	48.8	18.3	96	4.9	0.935	15.6	433	9.2	2.18	0.65
7	30	1.75	61.0	19.9	73	5.0	0.937	27.6	960	4.8	0.86	0.93
8	35	1.60	77.6	30.4	85	5.4	1.050	22.7	9300	20.0	4.19	5.84
9	36	1.8	64.7	20.0	92	5.6	0.964	20.4	973	8.0	1.82	1.12
10	39	1.65	71.3	26.3	83	5.4	1.04	18.7	7483	3.8	0.79	0.87
11	41	1.61	66.3	25.5	95	4.9	1.044	17.7	4808	15.0	3.52	3.37
12	47	1.61	64.3	24.8	90	5.1	0.962	30.2	1973	8.0	1.78	1.72
13	47	1.50	65.9	28.8	92	5.1	0.858	39.4	8417	14.0	3.18	1.49
14	48	1.78	73.9	23.3	84	5.5	1.108	32.0	2378	4.0	0.83	1.00
15	49	1.60	66.2	25.8	90	5.6	0.989	26.7	5506	7.5	1.67	3.26
16	54	1.65	62.8	23.0	85	5.6	0.959	34.1	2448	4.0	0.84	0.87
17	54	1.72	97.8	33.0	105	6.1	1.165	34.7	12855	6.0	1.55	6.87
18	56	1.67	57.8	20.7	98	5.5	0.742	47.3	12108	9.0	2.18	0.75
19	56	1.51	64.3	28.2	93	5.1	0.863	36.2	12748	5.0	1.15	3.20
20	57	1.70	71.6	24.7	90	6.4	0.979	31.0	1728	6.0	1.33	0.56
21	57	1.72	74.0	25.0	96	6.3	0.970	52.3	4859	9.0	2.13	1.13
22	59	1.55	60.2	25.0	89	5.1	1.084	23.6	5328	4.0	0.88	1.37
23	59	1.58	84.5	33.6	89	5.5	0.961	33.3	12664	-	-	19.9
24	59	1.46	75.6	35.4	84	5.8	0.895	28.4	11418	13.0	2.69	2.43
25	59	1.58	93.3	37.3	91	5.7	0.869	18.7	8764	14.0	3.14	4.43
26	62	1.66	62.0	22.5	93	6.1	0.940	49.5	4699	7.0	1.61	5.30
27	62	1.55	64.0	26.5	81	5.8	0.993	33.5	16553	28.0	5.59	4.17
28	64	1.64	71.0	26.3	96	5.8	0.965	36.3	2635	7.0	1.66	0.84
29	66	1.52	57.3	24.8	92	5.4	0.784	52.4	7817	-	-	8.00
30	67	1.54	66.8	28.1	87	5.7	0.956	35.1	8698	-	-	3.00
31	68	1.63	83.6	31.5	91	6.3	1.083	31.5	5766	16.0	3.59	5.76
